# Ability of *Euscelidius variegatus* to Transmit Flavescence Dorée Phytoplasma with a Short Latency Period

**DOI:** 10.3390/insects11090603

**Published:** 2020-09-05

**Authors:** Luca Picciau, Bianca Orrù, Mauro Mandrioli, Elena Gonella, Alberto Alma

**Affiliations:** 1 Dipartimento di Scienze Agrarie, Forestali e Alimentari (DISAFA), University of Torino, I-10095 Grugliasco (TO), Italy; luca.picciau@unito.it (L.P.); bianca.orru@unito.it (B.O.); 2 Dipartimento di Scienze della Vita (DSV), University of Modena e Reggio Emilia, I-41125 Modena, Italy; mauro.mandrioli@unimore.it

**Keywords:** vector, acquisition by adults, latency period, transmission process, midgut, salivary glands, phytoplasma epidemiology

## Abstract

**Simple Summary:**

Phytoplasmas are a group of phloem-restricted phytopathogens that attack a huge number of wild and cultivated plants, causing heavy economic losses. They are transmitted by phloem-feeding insects of the order Hemiptera; the transmission process requires the vector to orally acquire the phytoplasma by feeding on an infected plant, becoming infective once it reaches the salivary glands after quite a long latency period. Since infection is retained for all of the insect’s life, acquisition at the nymphal stage is considered to be most effective because of the long time needed before pathogen inoculation. This work provides evidence for the reduced latency period needed by adults of the phytoplasma vector *Euscelidius variegatus* from flavescence dorée phytoplasma acquisition to transmission. Indeed, we demonstrate that adults can become infective as soon as 9 days from the beginning of phytoplasma acquisition. Our results support a reconsideration of the role of adults in phytoplasma epidemiology, by indicating their extended potential ability to complete the full transmission process.

**Abstract:**

Phytoplasma transmission takes place by insect vectors through an Acquisition Access Period (AAP), Latency Period (LP) and Inoculation Access Period (IAP). Generally, phytoplasmas are believed to be transmitted more efficiently by nymphs because they need a long LP to reach the salivary glands before becoming infective. The transmission can start from adults as well, but in this case a long LP may exceed the insect’s lifespan. However, previous evidence has indicated that adults can undergo a shorter LP, even though little knowledge is available regarding the phytoplasma temporal dynamics during this period. Here, we investigate the minimum time required by the phytoplasma to colonize the vector midgut and salivary glands, and finally to be inoculated into a plant. We used the leafhopper *Euscelidius variegatus* to investigate the life cycle of flavescence dorée phytoplasma (FDP). Phytoplasma-free *E. variegatus* adults were left on broad beans (BBs) infected with FDP for an AAP of 7 days. Subsequently, they were individually transferred onto a healthy BB for seven different IAPs, each one lasting 24 h from day 8 to 14. Molecular analyses and fluorescence in situ hybridization were performed for FDP detection. FDP was found in the leafhopper midgut from IAP 1 with an infection rate reaching 50%, whereas in the salivary glands it was found from IAP 2 with an infection rate reaching 30%. FDP was also detected in BBs from IAP 4, with infection rates reaching 10%. Our results represent an important step to further deepen the knowledge of phytoplasma transmission and its epidemiology.

## 1. Introduction

Phytoplasmas are phloem-limited, wall-less bacteria of the class Mollicutes, associated with diseases affecting over one thousand cultivated and wild plant species [1]. Phytoplasma diseases in general are known and described throughout almost the entire world, although the different groups and subgroups are not evenly distributed geographically [2]. They are associated with many plants, both herbaceous and woody [3]; the symptoms attributable to these pathogenic agents are usually flower malformation (virescence, phyllody), yellowing, stunting, growth aberration, and decline [4]. Their relevance in agriculture has increased over the last few decades, and many diseases are now identified as related to phytoplasmas. In Europe, some of the most economically important diseases are grapevine yellows (flavescence dorée, FD; bois noir, BN); fruit-tree phytoplasmoses (apple proliferation, AP; pear decline, PD; European stone fruit yellows, ESFY); and stolbur (STOL) [5].

A special trait of the phytoplasma life cycle is their obligate interaction with plant and insect hosts; however, such a multi-actor interplay is still poorly investigated. In particular, understanding of the transmission process from vectors to plants is an essential step to figure out the epidemiology of the associated diseases [6], in order to develop suitable protection strategies. Phytoplasmas are transmitted in nature by hemipteran phloem-feeding insects belonging to the families Cicadellidae (leafhoppers), Cixiidae (planthoppers), and Psyllidae (psyllids) [7]. More than 75% of the known leafhopper vector species are included in the Cicadellidae subfamily Deltocephalinae; however, a number of vector species are also present within other subfamilies [8,9].

Phytoplasma transmission occurs by insect vectors through a set of peculiar phases, according to the persistent-propagative modality. The Acquisition Access Period (AAP) is the first one, which occurs when an insect feeds on an infected host plant. Concerning the life stage, nymphs are more efficient than adults in acquiring phytoplasmas [10] and show a longer period of infectivity before insect death, thus increasing phytoplasma spread. However, first-instar nymphs of *Macrosteles fascifrons* (Stål) and *Euscelidius variegatus* (Kirschbaum) are less likely to acquire phytoplasmas than older nymphs and adults, probably because of their short stylets [11,12]. Likewise, first-instar nymphs of *Scaphoideus titanus* Ball do not feed into the phloem of grapevine [13] and therefore are unable to acquire the phytoplasmas.

A Latency Period (LP) or incubation is needed for vectors before being able to transmit phytoplasmas to plants. At the end of the LP, the last phase takes place, called the Inoculation Access Period (IAP). During the LP, phytoplasmas cross the midgut epithelium to reach the hemolymph and actively multiply, colonizing the insect body, including the salivary glands [9,14]. The LP varies from a minimum of 12–14 days—in *Circulifer tenellus* (Baker) and beet leafhopper-transmitted virescence (BLTV) phytoplasma, and in *S. titanus* and FD phytoplasma (FDP)—to more than 21 days or even over a month in other cases [4,15,16,17,18,19,20]. The duration of the LP is characteristic for each vector–phytoplasma association. For example, differences in the infection temporal dynamics were demonstrated for *E. variegatus*, a lab vector, infected at the same time by FDP and Chrysanthemum Yellows phytoplasma (CYP) [21]. Differences in LP duration—i.e., in the time required to reach the insect’s salivary glands—are related to the host range width of different phytoplasmas, and to the co-evolution between insects and phytopathogens. Indeed, newly established associations may result in high immune activation in the insect midgut and hemolymph [22,23]. Even though the AAP may take place both in juveniles and adults [19], acquisition by nymphs can be more effective for the spread of phytoplasmas in the field when the LP is long, since acquisition by adults can result in a short period of infectivity before insect death [24]. Nonetheless, limited knowledge is available concerning the real LP occurring in adults, even though previous evidence has indicated that when acquisition occurs in adults, a shorter LP is needed, suggesting that the role of field specimens that acquire as adults may not be negligible [19].

The aim of this research was to assess the temporal dynamics of the phytoplasma transmission process, starting the AAP with adult specimens, identifying the minimum period required for phytoplasma to infect the insect midgut and salivary glands, and the shortest LP with successful transmission that can be recorded. For this purpose, we used an experimental pathosystem composed of *E. variegatus* as a vector, the broad bean as a host plant, and FDP [25].

## 2. Materials and Methods

### 2.1. Insect and Plant Material

A healthy colony of *E. variegatus* was maintained under laboratory conditions on oat plants (*Avena sativa* L., cv Aveny) in a climatic chamber at 25 °C, photoperiod 16 L:8 D, in order to obtain phytoplasma-free specimens. Insects were bred until adult stage, and newly emerged adults (less than 24 h old) were used for transmission trials. A PCR assay was performed on a batch of 10 randomly chosen specimens from the colony to confirm phytoplasma absence prior to transmission tests. Broad beans (*Vicia faba* L. cv. Aguadulce, BBs) infected with FDP were obtained by exposing seedlings to *E. variegatus* specimens infected with FD-C phytoplasma (16SrV-C) [26]. Healthy broad bean seedlings were grown in screen houses separated from the insect rearing space and other phytoplasma sources.

### 2.2. Transmission Experiments

Transmission trials were set up in a climatic chamber (T = 25 °C, RH = 75%, photoperiod 16 L:8 D). Newly emerged adults of *E. variegatus* were caged on BB plants previously infected with FDP and checked for infection by quantitative PCR [25,26,27], for the AAP. The acquisition access period lasted 7 days.

**Figure 1:**
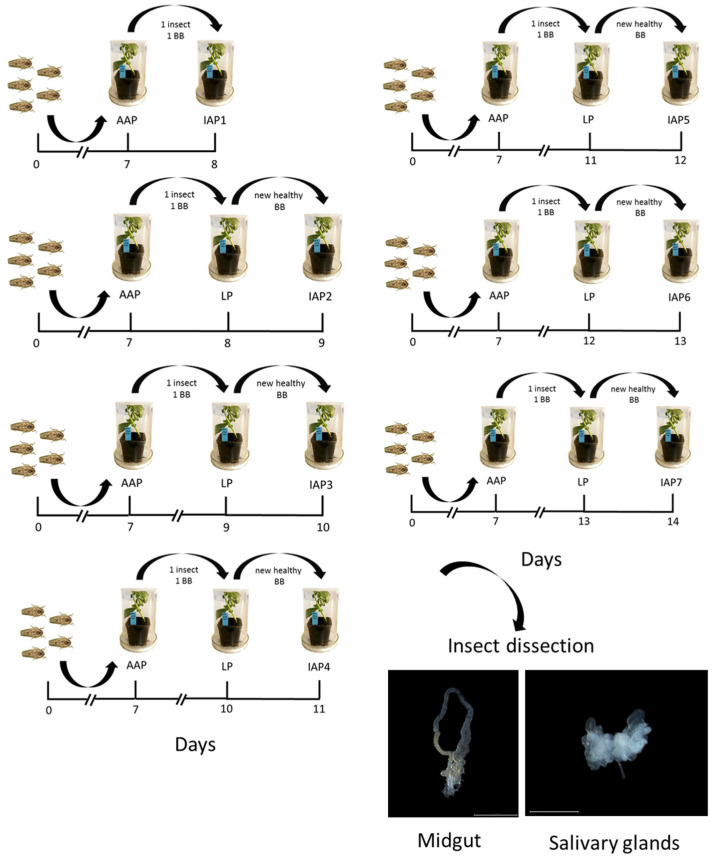
Experimental procedure followed for flavescence dorée phytoplasma (FDP) transmission trials. Adult *E. variegatus* specimens were exposed to an FDP-infected broad bean (BB) for an Acquisition Access Period (AAP) (day 0–7), then individually caged with a BB seedling for Latency Periods (LPs) of different durations (0 to 6 days). Finally, insects were moved onto a new healthy plant to perform a 24 h Inoculation Access Period (IAP) 1 to 7 days after the end of the AAP (IAP 1–7) (*n* = 40). At the end of the experiment (from day 8 for IAP 1 to day 14 for IAP 7), insects were collected and dissected to isolate the midgut and salivary glands for molecular or fluorescence in situ hybridization (FISH) analyses.

After the AAP, insects were immediately used to perform seven different IAPs, called IAPs 1–7, each one corresponding to the following 1 to 7 days and lasting 24 h. Specifically, at day 8, 40 adults of *E. variegatus* (20 males and 20 females) were sorted out and individually placed on a single healthy potted BB seedling caged inside a Plexiglas cylinder (h = 20 cm; diameter = 12 cm) for 24 h to perform IAP 1. The same procedure was used to perform IAP 2, but each insect was first transferred onto a single healthy BB seedling for a one-day LP at day 8, and then moved onto another healthy BB seedling to perform IAP 2 at day 9. The scheme was repeated for the following IAPs, increasing the LP on the same BB by one day every time until IAP 7 at day 14 (Figure 1). Therefore, for each IAP, 40 *E. variegatus* adults were employed to inoculate 40 BBs for 24 h, after a variable LP performed on another 40 plants (with the exception of IAP 1, which had no LP). At the end of every IAP, insects were collected to isolate the midgut and salivary glands, using sterile forceps under a stereomicroscope (Figure 1). Dissected tissues were stored at –20 °C until use for molecular analyses to detect FDP presence. Inoculated plants were treated with an insecticide (Etofenprox, TREBON^®^ UP, SIPCAM ITALIA S.p.A., Milan, Italy; 0.5 mL/L) and kept inside an insect-proof cage, in a climatic chamber, for three weeks from the beginning of phytoplasma inoculation. At the end of this period, the appearance of symptoms was recorded, and leaf vein samples were collected from all plants for molecular analyses. The same procedure was repeated for a second experiment, in order to perform fluorescence in situ hybridization (FISH) analysis. In this experiment, we selected only those IAPs where FDP colonization of salivary glands was previously observed by molecular diagnosis. Ten adults (five males and five females) were used for each IAP in this experiment. 

### 2.3. DNA Extraction and Quantitative Real-Time PCR Analysis

Total DNA extraction was performed separately from salivary glands and midguts dissected from single insects used in transmission experiments and from inoculated BBs. A protocol suitable for DNA extraction from leafhoppers was used for *E. variegatus* organs [28], with the following modification: DNA was resuspended in 20 μL of TE buffer, pH 8. Plant DNA was extracted according to the DNeasy Plant Mini Kit protocol instructions (Qiagen, Milan, Italy), from leaf tissue previously grounded with liquid nitrogen in a sterile mortar. 

Total DNA from insects and BBs was then submitted to quantitative real-time PCR (qPCR), in order to measure the presence and concentration of phytoplasma genome units (=cells) in those tissues. A CFX Connect ^TM^ Real-Time PCR Detection System (Bio-Rad, Richmond, CA, USA) was used with SsoAdvanced ^TM^ Universal SYBR ^®^ Green Supermix (Bio-Rad). Reactions targeting the 16S rRNA gene of group 16SrV phytoplasmas were carried out on all samples by using the fAY/rEY primer pair [29,30], with the conditions described by Galetto et al. [31]. The average FDP Genome Units (GU)/µL was calculated by dividing 16S rRNA gene copy numbers by two, because this gene is estimated to be in double copy in the genome of phytoplasmas [32]. The obtained values were then multiplied by the final elution volume (µL) of extracted DNA, i.e., by 20 (for insect organs) or by 100 (for plant samples), in order to obtain the absolute FDP GU/sample. An additional qPCR targeting the insect’s 18S rRNA was performed on gut and salivary gland samples, with the aim to normalize the absolute phytoplasma concentration. Primers MqFw/MqRv were used according to Marzachì and Bosco [33]. Normalized phytoplasma GU/sample was calculated per picogram of insect 18Sr RNA gene, whereas phytoplasma GU/sample from BBs was related to 100 milligrams of leaf. Serial dilutions of PCR-amplified 16S rRNA gene of FDP cloned with the pGEM^®^-T Easy Vector Cloning Kit (Promega, Milan, Italy) were used to obtain standard curves. The detection limit was calculated as the lowest concentration of cloned amplicons used for standard curves that was successfully amplified and corresponded to 1.0 × 10^0^ FDP GU.

### 2.4. Fluorescent In Situ Hybridization (FISH)

*Euscelidius variegatus* midguts and salivary glands were obtained from adults used to perform those IAPs where qPCR analysis highlighted the presence of FDP in the salivary glands. Whole mount FISH analyses were then carried out on dissected tissues from these insects. Hybridizations were conducted with probe ph1298, specifically targeting group 16SrV phytoplasmas, labeled with Cy5 (indodicarbocyanine, absorption/emission at 650/670 nm), as described by Lessio et al. [34]. Moreover, insect tissues were stained with DAPI (4′,6-diamidino-2-phenylindole, absorption/emission at 358/461 nm). Dissected organs were fixed for 2 min at 4 °C in 4% paraformaldehyde and washed in PBS. All hybridization experiment steps were performed following Gonella et al. [35]. After hybridization, the samples were mounted in antifading medium and then observed in a laser scanning confocal microscope SP2-AOBS (Leica Microsystems Srl, Buccinasco (MI), Italy).

### 2.5. Statistical Analysis

Statistical analyses were performed with SPSS Statistics 26^®^ (IBM Corp. Released 2016, Armonk, NY, USA). Each single insect and the corresponding BB were considered as a separate replicate to calculate the FDP infection rate, infectivity rate and transmission rate corresponding to each IAP. Infection rates were expressed as the number of FDP-positive midguts, midguts + salivary glands, and BBs, respectively, as a proportion of the total number of samples (*n* = 40). The infectivity rates for *E. variegatus* were expressed as the number of infective insects (midguts + salivary glands) as a proportion of the infected ones (at least midgut infected). The transmission rates were expressed as the percentage of infective insects (with FDP-positive midguts + salivary glands) that corresponded to an infected BB. Infection, infectivity and transmission rates were compared using a generalized linear model (GLM) with a binomial probability distribution—with positive samples marked as “1”, and negative samples as “0”—and a Bonferroni post hoc test (*p* < 0.05). The average concentrations of phytoplasma cells corresponding to different IAPs were compared using one-way analysis of variance (ANOVA), with separation of means by Tukey’s test (*p* < 0.05) when variance homogeneity was satisfied (Levene test, *p* < 0.05).

## 3. Results

Healthy *E. variegatus* adults were used for the AAP on 10 FD-infected BBs, in which the phytoplasma concentration ranged from 1.16 × 10^4^ to 2.96 × 10^5^ FDP GU/sample, with an average load of 9.71 × 10^4^ FDP GU/sample. The results of qPCR tests on samples used for the transmission trials are presented in Table 1. Males and females were considered as a single group as no difference was found among the results from each sex in the same IAP. The presence of FDP in the leafhopper midgut was detected from IAP 1 at day 8, and the infection rate in these organs reached 50%, whereas in the salivary glands FDP was detected from IAP 2 at day 9, with an infection rate gradually increasing and reaching its peak with 30% at IAP 7. As far as the BBs are concerned, FDP was detected from IAP 4 at day 11 with an infection rate reaching a maximum of 10% at day 14 (IAP 7). No significant difference between IAP levels was found by the GLM analysis either in the insect midguts or in BBs (*E. variegatus*: midguts χ^2^ = 5.928; df = 6; *p* = 0.431; BBs: χ^2^ = 10.819; df = 6; *p* = 0.094). In contrast, in salivary glands a significant increase in infection rates was recorded between IAP 1–4 groups and IAP 6 and 7 groups (χ^2^ = 54.753; df = 6; *p* < 0.001) (Table 1). No insect with FD-positive salivary glands and negative midgut was found. 

**Figure 2:**
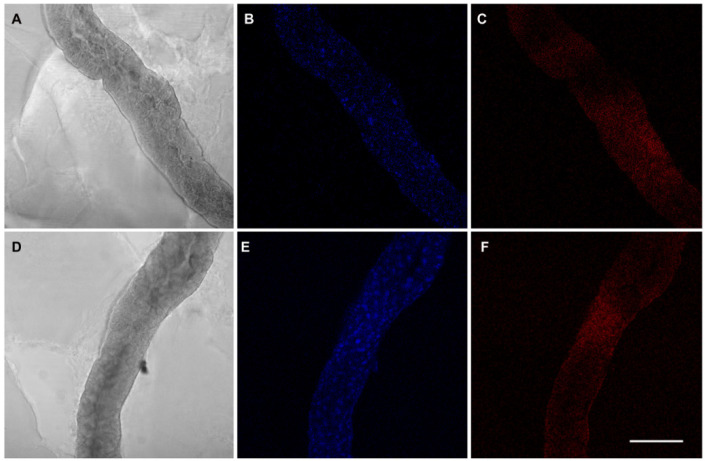
Whole mount fluorescence in situ hybridization on midgut samples from *E. variegatus* individuals after an AAP as adults. Exemplificative micrographs show the results corresponding to IAP 2 (**A**–**C**) and IAP 4 (**D**–**F**). Interferential contrast micrographs of the midguts are shown in (**A**) and (**D**); 4′, 6-diamidino-2-phenylindole (DAPI) staining of midgut tissues is reported in (**B**) and (**E**); hybridization with the 16SrV phytoplasma-specific probe is shown in (**C**) and (**F**). Bar = 100 μm.

**Figure 3:**
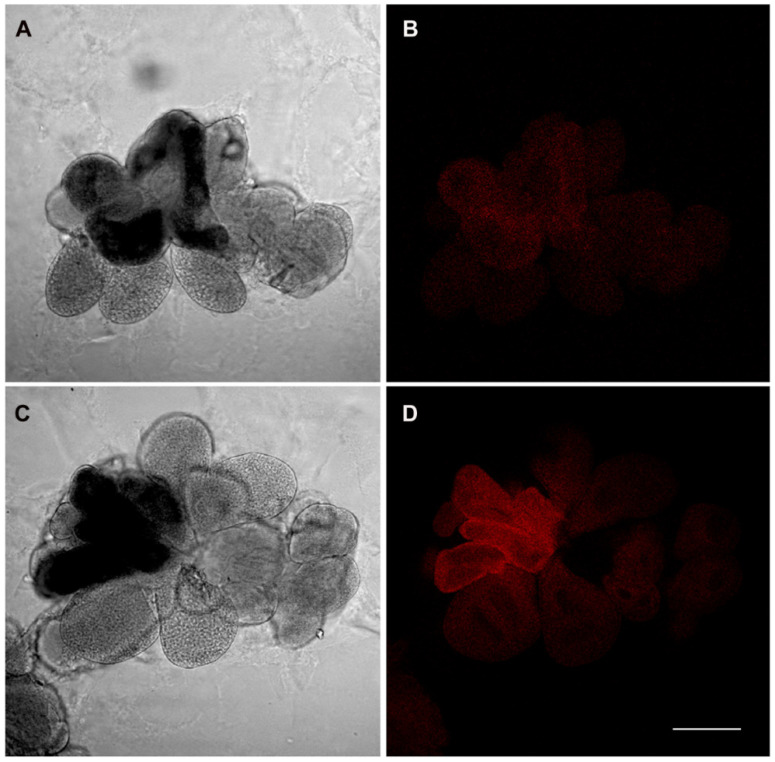
Whole mount fluorescence in situ hybridization on salivary glands from *E. variegatus* individuals after an AAP as adults. Exemplificative micrographs show the results corresponding to IAP 2 (**A,B**) and IAP 4 (**C,D**). Interferential contrast micrographs of the salivary glands are shown in (**A**) and (**C**), while hybridization with the 16SrV phytoplasma-specific probe is shown in (**B**) and (**D**). Bar = 150 μm.

The *E. variegatus* infectivity rates ranged from 24% at IAP 2 to a maximum of 63% at IAP 7, even though GLM analysis did not indicate significant differences (χ^2^ = 9.751; df = 5; *p* = 0.083) (Table 2). FISH experiments confirmed the presence of phytoplasmas in the insect midgut (Figure 2) and salivary glands (Figure 3) starting from IAP 2, consistently with qPCR results. 

The ratio between positive BBs and infective leafhoppers (transmission rate) was 17% at IAP 2 reaching 33% at IAP 7, again with no significance increment according to GLM (χ^2^ = 1.015; df = 3; *p* = 0.798) (Table 2). Furthermore, all of the FD-positive BBs were related to positive insects. One symptomatic plant was observed at IAP 4, and symptoms were recorded through the following IAP levels (1 out of 2 at IAP 5, 1 out of 3 at IAP 6, and 2 out of 4 at IAP 7), whilst no symptoms were observed in plants used for IAP 1–3. All symptomatic plants tested positive by qPCR.

**Figure 4:**
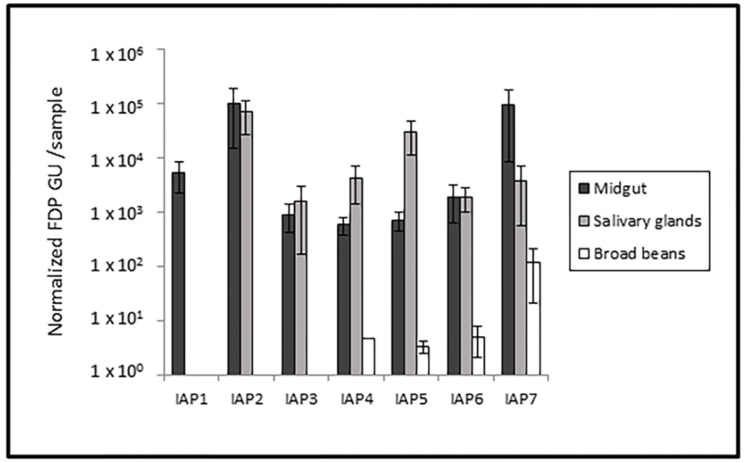
Average concentration of phytoplasma cells in midgut and salivary gland samples from FDP-positive *E. variegatus* corresponding to different IAPs, as well as in inoculated broad beans. Bars indicate the standard error.

The average concentration of phytoplasma cells ranged from 5.78 × 10^2^ to 9.86 × 10^4^ FDP GU/sample in the leafhopper midgut, from 1.60 × 10^3^ to 6.87 × 10^4^ FDP GU/sample in the salivary glands, and from 3.29 × 10^0^ to 1.16 × 10^2^ FDP GU/100 mg of plant tissue in inoculated BBs (Figure 4). In all sample groups, the IAP factor was not significant according to ANOVA (*E. variegatus*: midguts df = 6, 109; F = 0.957; *p* = 0.458; salivary glands df = 5, 37; F = 0.904; *p* = 0.489; BBs: df = 2, 6; F = 1.793; *p* = 0.245).

## 4. Discussion

The present study provides evidence for the first time that *E. variegatus* is able to acquire and transmit FD-C phytoplasmas from infected BBs during the adult stage. This vector is routinely used to maintain FDP in broad beans under laboratory conditions [26] for investigating the details of the FDP cycle [25]. However, most of the data regarding the transmission of FD-related phytoplasmas by adult leafhoppers have been previously inferred from the *S. titanus*–FDP association. In *S. titanus*, nymphs from the third instar can acquire phytoplasmas by feeding on an infected host plant, and transmit them at the adult stage after 21 days (AAP + LP) [36,37,38,39,40,41]. Nonetheless, Galetto et al. [42] demonstrated that *S. titanus* is able to acquire FD-C phytoplasmas from infected broad beans as an adult as well. Recently, Alma et al. [19] highlighted that in *S. titanus* inoculation may take place in a shorter time (14 days) on broad beans under laboratory condition after an AAP as an adult.

In our experiments, adult *E. variegatus* became potentially infective after as little as 9 days from the beginning of the AAP, when FDP was first detectable in the salivary glands. Nonetheless, the observed temporal dynamics of phytoplasma colonization of salivary glands indicated that a relatively longer period could be required for FDP cells to stably settle in salivary glands, supporting an effective inoculation into plants, as demonstrated by the significant difference in the infection rates for these organs throughout the different performed IAPs. Consistently, successful inoculation occurred only at day 11, which still represented a very short LP. Under the same experimental conditions, nymphs of *E. variegatus* were previously demonstrated to be unable to transmit FDP to broad beans before 28 days (AAP + LP), even though the pathogen was detectable in the whole insect body as soon as 14 days from the beginning of the AAP [21]. The same vector successfully transmitted another phytoplasma, namely CYP, after an LP of 17–28 days starting from nymphs [21,43]. Since the time required for the colonization of *E. variegatus* salivary glands by FDP was reported to be slower at the nymphal stages than the time required by the co-evolved CYP [21], we can speculate that the LP occurring in adults may be even shorter for CYP. Furthermore, the high FDP titer and infection rate detected in the midgut as early as day 8 suggest an effective colonization of the intestine tissues by the phytoplasma, which reinforces this fast infection dynamics. Furthermore, the phytoplasma concentration in the insect vector was higher than what was obtained in *S. titanus* [19]. Moreover, previous reports indicated that, in nymphs of *E. variegatus*, more than 30 days are necessary for FDP to reach a concentration comparable to the titer recorded here from IAP 1 [21]. The long LP required by FDP in juveniles could be related to immune activation, especially in the hemocytes [23]. Since the number of circulating hemocytes in the hemolymph may be highly variable according to the insect life stage [44], a different immune response may be observed during phytoplasma infection in nymphs and adults of *E. variegatus*, allowing a faster colonization of adult individuals. 

The phytoplasma load in the salivary glands did not vary significantly over a period of 14 days (7 days AAP + 7 days LP), indicating that in such a short time only limited pathogen multiplication takes place inside the leafhopper’s body. Rather, a high phytoplasmal load is ingested by insects during the AAP. In case of AAP performed on nymphs, the minimum reported time required is 1 h for *E. variegatus* and Italian clover phyllody phytoplasma [45], while Bosco and Marzachì [24] reported 4 h for *Macrosteles quadripunctulatus* (Kirschbaum) and *E. variegatus* to acquire CYP. However, the AAP reported in the literature for most phytoplasma transmissions performed at nymphal instar ranges from a few to several days [24]. The LP also shows a wide range, from 12 days for *C. tenellus* and BLTV to over a month in other cases, as reviewed by Marzachì et al. [4]. Temperature was shown to influence the phytoplasma multiplication in the vector body [46], even if there is sometimes no clear correlation between phytoplasma titer and LP in the vector [47]. For example, high titers of the corn stunt spiroplasma are more rapidly reached in *Dalbulus maidis* (DeLong & Wolcott) when multiplication starts from a higher load [48]. Therefore, the relatively high titer detected in the *E. variegatus* salivary glands might be due to a high level of FDP present in the BBs used for AAP.

It is generally believed that gender has an influence on phytoplasma acquisition and transmission [8,10,11,49,50], since females are apparently more efficient than males. For example, sex-related differences in transmission efficiency were reported in vectors infected with phytoplasmas in the Aster Yellows group, probably because of their different behavior [4]. Our results seem to be in contrast to these previous studies since no difference between sexes was found, probably due to the very short LP needed by the adult leafhoppers. 

The average percentage of phytoplasma-inoculated BBs was lower in our experiments than in a previous study involving *S. titanus* adults and FDP [19]; however, in that work, groups of five leafhoppers inoculated a single plant, increasing the chance of successful transmission. Conversely, taking into consideration the transmission rates, our results showed high efficiency in inoculating the BBs. Moreover, in our experiments the FDP load recorded in the plant was similar to the phytoplasma concentration reported by Alma et al. [19]; in both cases, this was low when compared with previous work [51]. Consistently, symptoms were observed only in some of the positive plants, probably because in some BBs the inoculated phytoplasma load was not sufficient to induce symptoms. This was likely due to the limited number of infected leafhoppers released on each plant during the IAP, one in the case of *E. variegatus* and five for *S. titanus* [19], suggesting that potential transmission efficiency could be even higher under different experimental conditions.

## 5. Conclusions

Although the relationship between phytoplasma transmission and insect vectors has been widely studied to date, new insights are still arising to fill the remaining gaps. The ability of *E. variegatus* to acquire and transmit FDP during the adult stage with a short LP under laboratory conditions represents an important integration into the knowledge of phytoplasma transmission at the ecological and biological levels. These results complete and clarify what was previously reported by Alma et al. [19] for *S. titanus*, and they represent a relevant step to better explain phytoplasma epidemiology. Finally, increased awareness concerning the phytoplasma life cycle in the vector could support the implementation of new disease control methods.

## Figures and Tables

**Table 1:** Rate of flavescence dorèe phytoplasma infection in *E. variegatus* midgut and salivary glands after an acquisition access period at the adult stage, and in broad beans inoculated by single specimens. The number of infected samples for each group is indicated (*n* = 40). Different letters indicate significantly different results according to generalized linear model (GLM) analysis (*p* < 0.001).

Inoculation Access Period (IAP)	No. Infected Midguts	Midgut Infection Rate ± SE ^1^	No. Infected Midguts + Salivary Glands	Midgut + Salivary Glands Infection Rate ± SE ^1^	No. Infected BB	Broad beans Infection Rate ± SE ^1^
1	20	0.50 ± 0.08 n.s. ^2^	0	0.00	0	0.00
2	17	0.425 ± 0.08 n.s. ^2^	4	0.10 ± 0.04 ^a^	0	0.00
3	18	0.45 ± 0.08 n.s. ^2^	6	0.15 ± 0.06 ^ab^	0	0.00
4	12	0.30 ± 0.07 n.s. ^2^	6	0.15 ± 0.06 ^ab^	1	0.03 ± 0.02 n.s. ^2^
5	13	0.325 ± 0.07 n.s. ^2^	5	0.12 ± 0.05 ^ab^	2	0.05 ± 0.03 n.s. ^2^
6	17	0.425 ± 0.08 n.s. ^2^	10	0.25 ± 0.07 ^b^	3	0.08 ± 0.04 n.s. ^2^
						
7	19	0.475 ± 0.08 n.s. ^2^	12	0.30 ± 0.07 ^b^	4	0.10 ± 0.05 n.s. ^2^

^1^ Standard Error according to the GLM output (SPSS Statistics); ^2^ Not significant.

**Table 2:** Infectivity and transmission rates recorded for adult *E. variegatus* specimens after an AAP at the adult stage. The infectivity rates are calculated as the ratio between the insects with infected midgut + salivary glands (infective) and those with infected midgut (infected), as shown in Table 1. The transmission rate to broad beans was calculated as the ratio between the number of infected plants and infective *E. variegatus* specimens, as indicated in Table 1.

IAP	*E. variegatus* Infectivity Rate ±SE ^1^	*E. variegatus* Transmission Rate to BB ±SE ^1^
1	Not Applicable (NA)	NA ^3^
2	0.24 ± 0.11 n.s. ^2^	NA ^3^
3	0.33 ± 0.11 n.s. ^2^	NA ^3^
4	0.50 ± 0.15 n.s. ^2^	0.17 ± 0.17 n.s. ^2^
5	0.38 ± 0.14 n.s. ^2^	0.40 ± 0.24 n.s. ^2^
6	0.59 ± 0.12 n.s. ^2^	0.30 ± 0.15 n.s. ^2^
7	0.63 ± 0.11 n.s. ^2^	0.33 ± 0.14 n.s. ^2^

^1^ Standard Error according to the GLM output (SPSS Statistics); ^2^ Not significant; ^3^ Not Applicable.
